# Official statistics and claims data records indicate non-response and recall bias within survey-based estimates of health care utilization in the older population

**DOI:** 10.1186/1472-6963-13-1

**Published:** 2013-01-03

**Authors:** Matthias Hunger, Larissa Schwarzkopf, Margit Heier, Annette Peters, Rolf Holle

**Affiliations:** 1 Helmholtz Zentrum München, German Research Center for Environmental Health (GmbH), Institute of Health Economics and Health Care Management, Ingolstädter Landstr. 1, Neuherberg, 85764, Germany; 2 Helmholtz Zentrum München, German Research Center for Environmental Health (GmbH), Institute of Epidemiology II, Ingolstädter Landstr. 1, Neuherberg, 85764, Germany

**Keywords:** Health care utilization, Self-report, Validity, Survey, Response bias, Recall bias, Claims data

## Abstract

**Background:**

The validity of survey-based health care utilization estimates in the older population has been poorly researched. Owing to data protection legislation and a great number of different health care insurance providers, the assessment of recall and non-response bias is challenging to impossible in many countries. The objective of our study was to compare estimates from a population-based study in older German adults with external secondary data.

**Methods:**

We used data from the German KORA-Age study, which included 4,127 people aged 65–94 years. Self-report questions covered the utilization of long-term care services, inpatient services, outpatient services, and pharmaceuticals. We calculated age- and sex-standardized mean utilization rates in each domain and compared them with the corresponding estimates derived from official statistics and independent statutory health insurance data.

**Results:**

The KORA-Age study underestimated the use of long-term care services (−52%), in-hospital days (−21%) and physician visits (−70%). In contrast, the assessment of drug consumption by postal self-report questionnaires yielded similar estimates to the analysis of insurance claims data (−9%).

**Conclusion:**

Survey estimates based on self-report tend to underestimate true health care utilization in the older population. Direct validation studies are needed to disentangle the impact of recall and non-response bias.

## Background

According to population forecasts, one third of the German resident population will be aged 65 or older in 2050 [1]. Thus, studies on resource utilization in the elderly population will gain in importance for reasons of financing and health services research. Health insurance claims data are generally considered to provide the most precise information on service utilization and costs [2]. In many countries, however, linking health surveys with administrative data is challenging if not impossible [3]. In Germany, for example, no single source exists for resource utilization data. Thus, researchers have to contact numerous payers and service providers to collect comprehensive data, making this approach costly and time-consuming in practice [4,5].

A common alternative to estimating service utilization are population-based surveys with retrospective self-reports. Although such methods are easy to apply in practice, the information obtained may be biased due to poor recall of past events [6]. Studies have shown that recall of resource utilization declines over time and that some components of healthcare utilization may be recalled better than others [2,7].

Another problem with survey research is the possible non-response bias, which occurs if there are different utilization rates between study participants and those who were invited but did not respond [8-10]. Direct assessment of non-response bias is only feasible if administrative data from both, survey participants and non-responders are available. In many countries, however, obtaining health care information about non-responders without their consent is not possible on account of national data protection legislation.

In Germany, several surveys on health care utilization in the elderly have been conducted in recent years [11-13]. However, given the above hurdles for linking individual survey data to administrative data and the prohibition on using non-responder data without consent, studies on the impact of recall and non-response bias are largely lacking. The second best alternative in evaluating the accuracy of estimates is to compare summary statistics from the survey with corresponding summary statistics from other sources such as official statistics and insurance data [14,15]. However, we know of no German study that has yet undertaken such a comparison. Evaluating potential biases is especially important for surveys in the older population where recall may be worse than in younger ages, and where non-response may be caused by illness or disability which in turn is a predictor of higher utilization levels [16].

The purpose of this study was to compare estimates of health care utilization calculated from the population-based German KORA-Age study with corresponding estimates obtained from official statistics and an independent statutory health insurance sample.

## Methods

### KORA-Age study

The KORA-Age study was conducted between December 2008 and October 2009 as a longitudinal study focusing on research into multimorbidity in the older population. The study design was based on ongoing studies from KORA research, a platform for population-based surveys and subsequent follow-up studies in the fields of epidemiology, health economics and health care research in Germany [17]. The KORA-Age study is a follow-up of all participants aged 65–94 in the MONICA/KORA surveys S1 to S4 conducted between 1984 and 2001. In these surveys, participants were randomly selected from population registries in the study region, comprising the city of Augsburg and its two surrounding counties in the federal state of Bavaria. Participation rates ranged between 79% and 67%. Details about study design, sampling method and data collection can be found elsewhere [17-19]. In total, 17,607 people participated in at least one of the four surveys. The KORA-Age study population is restricted to the subgroup of 9,197 subjects born in 1943 or earlier. Of these, 2,734 individuals had died, 45 had moved away and 427 refused to be contacted for any follow-up, resulting in 5,991 eligible people with known addresses.

The KORA-Age study combined two designs. First, a morbidity follow-up questionnaire covering major diseases and drug utilization was sent by post to all 5,991 eligible individuals. Second, a 30-minute telephone interview with more in-depth questions was conducted about 2 weeks after the return of the questionnaire, in which participants were also asked about their utilization of outpatient, inpatient and long-term care services. If a telephone interview was not possible, individuals were offered the chance to be interviewed at home. The questions could also be answered by a proxy (i.e. a family member or professional care giver) if the respondent was unable to participate.

In total, 4,127 interviews on resource utilization were performed (response 68.9%), of which 60 were performed at the participants’ home and 185 were proxy interviews.

### Assessment of resource use

Questions on health service utilization in the KORA-Age study covered inpatient services, outpatient services, drug utilization, and long-term care services according to the scope of compulsory long-term care insurance (LTCI) [20,21] The first three domains are covered by the Statutory Health Insurance (SHI) and the last by LTCI, which is a separate branch within the German social security system covering various community-based and institutional nursing care services. LTCI defines three care levels reflecting the applicant’s need for support in activities of daily living. Each level is connected to a fixed monthly tariff for community-living and institutionalized beneficiaries.

Long-term care utilization in the KORA-Age study was assessed by asking ‘Did you use services covered by the LTCI in the past 12 months?’ and, if ‘yes’, ‘Which care level are you assigned to?’.

For inpatient services, the number of hospital days was assessed by asking ‘Have you been hospitalized in the past 12 months?’ and, if ‘yes’, ‘How many days have you been hospitalized in the past 12 months?’. Outpatient services were assessed by asking the question, ‘How often did you see a physician (general practitioner or specialist) in the past 3 months?’. Finally, drug consumption was assessed in the postal questionnaire and covered the medications taken in the past 7 days including both prescribed and over-the-counter (OTC) drugs. Participants were asked to write down the exact name and central pharmaceutical number (PZN) for each medication. The PZN is a nationwide standardized identification number for proprietary medical products in Germany enabling a well-defined attribution of a pharmaceutical product including, for example, name, package size and defined daily dose (DDD).

### Comparative data

Data used for external comparison come from independent insurance claims data and official statistics.

The insurance data used for comparison refer to an excess cost study with dementia patients and non-demented control subjects conducted by Schwarzkopf et al. [22]. The corresponding sample was provided by AOK Bavaria, a large local SHI fund, from which we obtained permission to reuse the data in our analyses. It provided 2005 to 2007 claims data for its insured clients living in the Bavarian district of Middle Franconia who were aged 65 years and older in 2006. AOK is the leading SHI fund in that district, covering about 50% of the population in the respective age range. For the design of the above study, each insured person with diagnosed dementia was matched with four non-demented control subjects. As the objective of the present study was to estimate utilization rates for the general population, we recreated the original dementia prevalence in each 5-year age and sex group by deleting the supernumerous dementia patients per group. This led to a final insurance sample of 37,546 insured people. We analysed the 2006 claims data rather than the more recent 2007 data because the original matching of demented and non-demented individuals was based on the 2006 data and because using the 2007 data would result in losing claims data on individuals aged 65 years.

AOK claims data were used as a comparator for all four utilization domains in the KORA-Age study. We also compared estimates of long-term care utilization with published official reports that are freely available from the Bavarian Office for Statistics and Data Processing [23,24]. These reports provide information on the total number of LTCI beneficiaries and their care levels in Bavaria in 2009.

**Figure 1:**
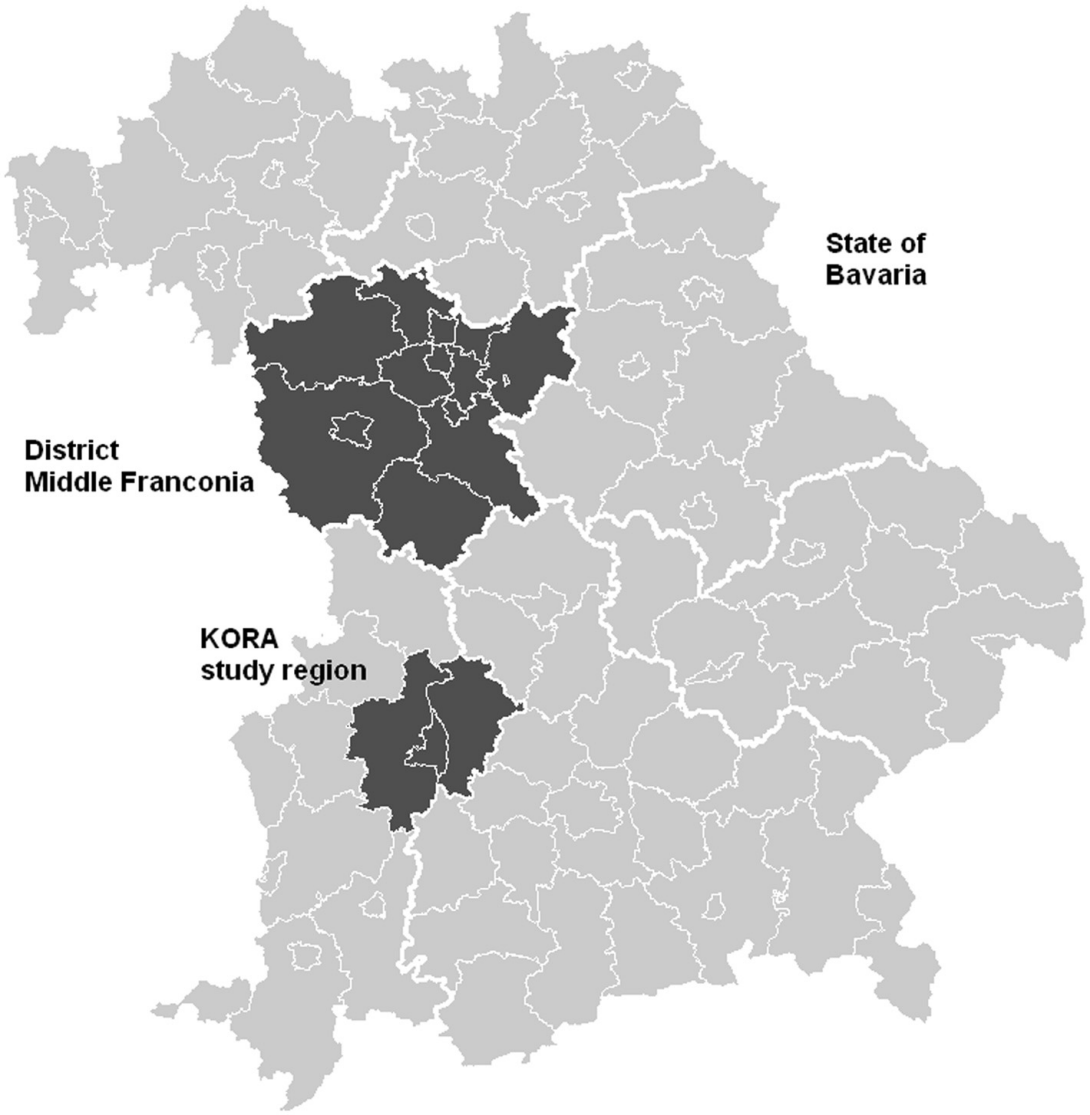
Location of Middle Franconia and the KORA study region in the state of Bavaria.

A comparison between utilization estimates from the above data sources requires the consideration of three major methodological differences between them. First, data were partly collected at different times: Whereas the majority of KORA-Age participants were assessed in 2009 and the official statistics on long-term care utilization also refer to 2009, the AOK claims data date back to 2006. Published statistics showed that, with the exception of long-term care use, utilization of health care services has increased during this 3-year period [25,26]. This was most pronounced for pharmaceuticals [27,28]. Second, although all data sources refer to people residing in Bavaria, several only focus on specific Bavarian counties or districts: Whereas the KORA-Age study was conducted in the city of Augsburg and its two surrounding counties, the insurance data comprise individuals from the district of Middle Franconia. The location of the different geographical entities in question is depicted in Figure 1. Third, the three data sources differ slightly with respect to the reference population they are targeting: Whereas the KORA-Age study was conducted in the general population with German nationality, the official statistics as well as the AOK claims data also include non-German individuals. Furthermore, given that AOK Bavaria is an SHI, the data do not comprise individuals insured with private health insurance companies traditionally chosen by the wealthier population – these make up less than 10% of the general population aged 65 and older.

To address the above methodological differences appropriately, we proceeded as follows. To examine potential differences between the geographical entities, we compared official statistics on the distribution of age, sex and morbidity in the KORA study region in 2009 and the district of Middle Franconia in 2006 with the respective statistics for the Federal State of Bavaria in 2006 and 2009. To adjust for the development of health expenditures between 2006 and 2009, we calculated domain-specific correction factors as follows. For inpatient and outpatient domains, we extrapolated the observed growth rates in the AOK claims data between 2006 and 2007 to the year 2009. For drug utilization, we applied a very thorough extrapolation method based on published national age- and anatomical therapeutic chemical (ATC)-specific growth rates in the consumption of DDD [27,28]. As a sensitivity analysis, we also displayed unextrapolated 2006 data within our tables. Given that utilization rates for long-term care remained stable between 2006 and 2009, we did not extrapolate 2006 values [23,24] . Finally, when comparing estimates from the KORA-Age study with those derived from the AOK claims data, we excluded all non-German individuals in the AOK sample and all KORA-Age subjects who were privately insured. Moreover, we restricted presentation of our results to individuals aged between 65 and 90 years because only nine KORA-Age participants were older than 90 years.

As AOK insures a higher proportion of individuals with lower socio-economic status compared with other health insurance funds and as this may be associated with higher health service use, we conducted another sensitivity analysis in which we only considered KORA-Age participants insured by the AOK. As the name of the health insurance fund was not assessed in the KORA-Age study, we used this information from the baseline surveys.

### Comparison of resource use components

For each domain and in each data source, we first calculated utilization rates stratified by 5-year age and sex groups and then calculated standardized rates using the sex and age distribution in Bavaria 2009 as a reference population.

#### Long-term care insurance

In the long-term care domain, we compared the estimates from the entire KORA-Age sample with the estimates derived from the official Bavarian statistics. For comparison with the AOK data, we excluded all KORA-Age subjects with private health insurance. We calculated the overall percentage of individuals entitled to LTCI services and their distribution over the three different care levels.

#### Inpatient services

In the inpatient domain we calculated the mean number of hospital days per individual. Information on hospital treatment in claims data includes all services provided by a hospital (inpatient services, day-care services, outpatient surgery and ambulatory emergency treatment). In contrast, the KORA-Age questionnaire exclusively targeted inpatient services, requiring at least one overnight stay. To ensure comparability, we identified inpatient hospital treatment from the claims data via billing and documentation characteristics.

#### Outpatient services

Considering the outpatient domain in the AOK data, we calculated the number of physician visits per year. It is noteworthy that these ‘visits’ do not necessarily require direct patient–physician interaction but also include repeat prescriptions. To obtain a comparable measure in the KORA-Age study, we extrapolated the number of visits assessed for one quarter to 12 months by multiplying it by 4.

#### Drug utilization

The comparison of drug utilization rates between a health survey and insurance data is not straightforward [29]. First, the KORA-Age study gives a 7-day ‘snapshot’ of the medication currently used by each participant, whereas the AOK data only provide information on which date a medical prescription was filled. Thus, it is challenging to determine over what time horizon individuals take the corresponding drug or even how many drugs are taken at the same time [30]. Second, insurance data only contain pharmaceuticals that are reimbursed by SHI and disregard the entire OTC sector, whereas participants in the KORA-Age study were asked to report any kind of medication, which also included OTC drugs. Third, drug utilization rates may be difficult to compare because of different package sizes.

In order to establish a sensible standard of comparison, our measure of interest was the number of drugs from different pharmaceutical groups for the treatment of major chronic conditions that individuals take at the same time. We restricted our analyses to drugs for chronic conditions because they are more comparable across the two data sources than drugs used as needed [29,31]. Moreover, chronic conditions require continuous pharmacological treatment; thus different package sizes and the reach of one single prescription are no longer relevant. The diseases considered in our analyses were those listed in the classification of medications for chronic conditions published by Lamers and van Vliet [32]. Their algorithm identifies chronic conditions on the basis of ATC codes of the medicaments typically used to treat them. For example, an individual is identified as having diabetes if she/he is taking drugs with ATC code A10A (insulins) or A10B (oral blood glucose-lowering drugs). We excluded OTC drugs from the KORA-Age study because these are not documented in insurance claims data. In order to have a time span in the AOK data that is comparable to the ‘snapshot’ character of the data in the KORA-Age study, we only considered prescriptions that AOK insurants filled during the second quarter of 2006. We chose this 3-month time span for two reasons. On the one hand, we wanted the reference time span to be as short as possible. On the other hand, it needs to be long enough to provide records on each drug used to treat a chronic condition [33]. As the largest package size covers a maximum of 3 months, a patient with a chronic condition has to fill a prescription at least once per quarter. The 3-month period has shown high sensitivity compared with shorter periods for capturing the use of medicines for chronic conditions [29,33].

In summary, the number of drugs taken to treat chronic conditions in the AOK claims data was calculated as follows. First, all drugs prescribed during the reference quarter were grouped according to the ATC groups defined by Lamers and van Vliet [32]. Second, drugs per therapeutic subgroup were only counted once even if they were prescribed more than once during the reference period. This is to ensure that subsequent prescriptions of two small packs are considered equivalent to the prescription of one big pack. This approach may lead to a conservative estimate for the number of drugs because it does not account for combination therapies of drugs within the same ATC subgroup. For example, an insured person with diabetes combining insulin and oral anti-diabetic agent therapy is assigned two different drugs (A10A and A10B respectively), whereas a patient combining two different oral anti-diabetic agents is only assigned one drug.

As exactly the same method was applied to the reimbursed drugs reported by the KORA-Age participants, possible underestimation for combination therapies only affects the level of drug consumption, not the comparison between the data sources.

### Comparison between individuals with and without diabetes

In addition to considering the overall level of health care utilization, it is also interesting to examine whether health surveys validly estimate differences between various groups of individuals, e.g. between those with and without a certain disease. We addressed this question using diabetes as an example because diabetes has a high prevalence in older adults and is known to be a significant economic burden to health care systems.

To ensure consistency in the identification of diabetes between survey and insurance data, we only considered subjects who received oral anti-diabetic drugs or insulin (ATC code A10). The alternative would have been to consider physician diagnoses in the claims data and self-reports in the survey data. However, we considered this approach to be more likely to be subject to bias as some people with physician-diagnosed diabetes may not report having the disease and documentation of diabetes by the physician may not always be complete [34].

We calculated the ratio of age- and sex-adjusted mean utilization rates (number of hospital days and number of physician visits per year) between individuals with and without diabetes in the KORA-Age study and compared this ratio with the respective estimate from the AOK insurance data. The mean utilization rates were estimated using a linear model adjusting for sex and age groups and holding these covariates fixed at the corresponding mean values of the Bavarian population in 2009.

## Results

### Similarity of the KORA study region and the district of Middle Franconia

To ensure that the KORA study region and the district of Middle Franconia reflect comparable populations, we contrasted demographic information derived from official Bavarian statistics for both regions with overall Bavarian figures. As presented in Table 1, the characteristics of the district of Middle Franconia resembled quite well those of the Federal State of Bavaria in 2006, which was also the case for the KORA study region in 2009.

**Table 1:** Official statistics on sociodemographics and morbidity: Comparison of KORA study region, district of Middle Franconia and the Federal State of Bavaria

**Variable**	**Comparison of Middle Franconia (MFr) and Bavaria (BY) in 2006**			**Comparison of KORA study region (KORA SR) and Bavaria (BY) in 2009**		
	**Quota (%) BY**	**Quota (%) MFr**	**Odds ratio**	**Quota (%) BY**	**Quota (%) KORA SR**	**Odds ratio**
**Sex**						
Male	41.9	41.7	1.00	42.8	42.7	1.00
Female	58.1	58.3	1.00	57.2	57.3	1.00
**Age group (years)**						
65-69	33.4	33.2	1.01	28.8	29.6	0.97
70-74	23.8	23.8	1.00	27.5	27.1	1.01
75-79	18.8	19.0	0.99	18.3	18.4	0.99
80-84	13.9	13.8	1.01	13.9	13.9	1.00
85-89	7.0	7.0	1.00	8.4	8.2	1.02
90+	3.2	3.2	1.00	3.0	2.8	1.07
Entitled to long-term care services*						
Yes	2.5	2.4	1.02	2.6	2.3	1.10
No	97.5	97.6	1.00	97.5	97.7	1.00
	**Mean BY**	**Mean MFr**	**Delta**	**Mean BY**	**Mean KORA SR**	**Delta**
Hospital discharges per 1000 inhabitants*	198	199	–1	213	210	3
Length per hospital stay (days)*	8.4	8.7	−0.3	7.8	7.6	0.2

Source: Bavarian Office for Statistics and Data Processing.

*Figures refer to the entire resident population (all age groups).

### Baseline characteristics of KORA-Age participants and AOK Middle Franconia insured people

Of the 4,127 KORA-Age participants interviewed by telephone, 2,015 (48.8%) were male. Mean age was 73.4 years (SD 6.1) with the oldest participant being 93 years old. The proportion of privately insured participants in the sample was 9.6%. In the AOK insurance sample, 36,875 individuals (98.2%) were of German nationality. Of those, 10,344 were male (28.1%), and the mean age was 79.8 years.

### Entitlement to long-term care services

Table 2 shows that the likelihood of being entitled to long-term care services – defined as any care level assignment – increased steadily with age. Irrespective of the chosen data source, it was about 10 times higher in the oldest age group compared with the youngest one. Across the entire age range, the AOK sample yielded the highest quota of beneficiaries, but the difference was small compared with the official statistics. The KORA-Age sample continuously indicated a significantly lower quota.

**Table 2:** Age- and sex-specific entitlement to long-term care services

		**Males**					**Females**					**Age/sex standard**
	**Age group (years)**	**65–69**	**70–74**	**75–79**	**80–84**	**85–90**	**65–69**	**70–74**	**75–79**	**80–84**	**85–90**	
**Entitlement to long-term care services**	KORA-Age*	1.2%	2.1%	6.4%	4.1%	13.6%	0.6%	1.6%	5.0%	12.7%	24.2%	5.0%
	AOK SHI fund 2006	3.8%	6.3%	9.2%	16.7%	28.1%	3.3%	4.7%	9.9%	23.4%	39.0%	10.5%
	Delta as a %	−68%	−67%	−30%	−75%	−51%	−82%	−67%	−50%	−46%	−38%	−52%
	KORA-Age^†^	1.0%	1.9%	6.0%	4.1%	13.1%	0.6%	1.6%	4.9%	12.2%	24.4%	4.9%
	Bavaria 2009	2.4%	4.2%	8.0%	14.8%	26.9%	2.1%	4.0%	9.5%	20.4%	39.1%	9.2%
	Delta as a %	−59%	−56%	−25%	−72%	−51%	−73%	−60%	−48%	−40%	−38%	−47%

*Excluding KORA-Age participants with private health insurance.

^†^Including KORA-Age participants with private health insurance.

**Figure 2:**
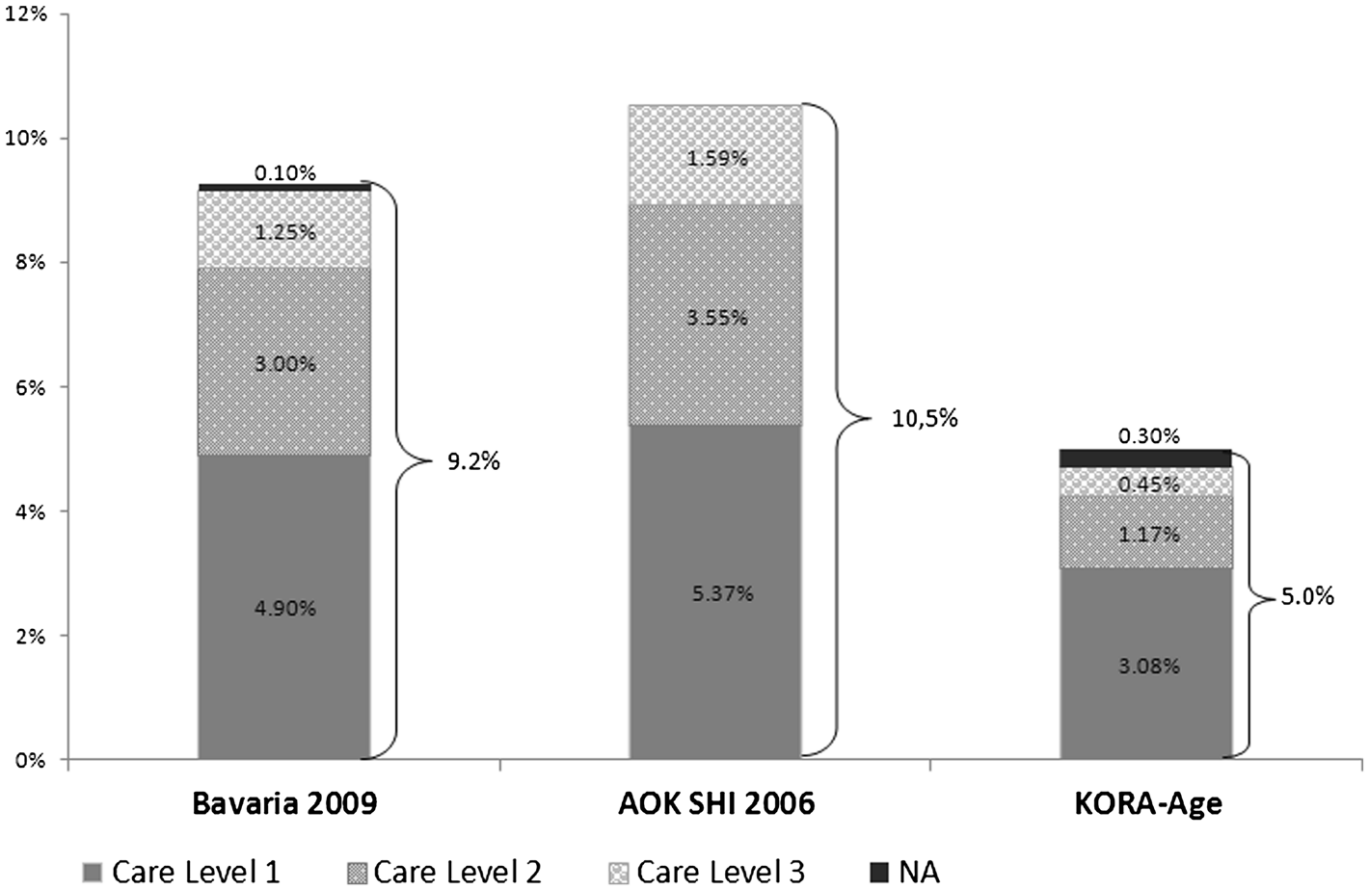
**Percentage of individuals entitled to long-term care services.** Percentage of individuals entitled to long-term care services; age- and sex-standardized for the Bavarian resident population NA: Not applicable; Care level unknown or application for long-term care is under consideration but not yet decided.

Figure 2 describes the distribution of care levels within the three data sources, standardized for the Bavarian resident population of 2009. According to official statistics and AOK data, around 50% of beneficiaries were mildly dependent (care level 1), around 33% were moderately dependent (care level 2) and around 15% were severely dependent (care level 3). Within the KORA-Age sample, around 66% were assigned to care level 1, around 25% to care level 2 and less than 10% to care level 3.

### Inpatient services

We compared self-reported inpatient days from the KORA-Age study with extrapolated AOK data to assess the volume of service use. As seen in Table 3, claims data generally indicated more inpatient days per capita than survey data. Irrespective of age, the difference between both data sources was more pronounced in male participants. Standardized for the Bavarian resident population, the volume of service utilization in the KORA-Age sample was around one fifth lower (3.4 versus 4.3 in-hospital days).

**Table 3:** Age- and sex-specific mean in-hospital days, physician visits and drugs for the treatment of chronic conditions

		**Males**					**Females**					**Age/sex standard**
	**Age group (years)**	**65-69**	**70-74**	**75-79**	**80-84**	**85-90**	**65-69**	**70-74**	**75-79**	**80-84**	**85-90**	
**Inhospital days**	KORA-Age	2.8	2.5	3.1	3.3	5.4	3.7	2.7	3.7	4.0	5.1	3.4
	AOK SHI fund 2009*	2.4	4.9	5.1	5.5	7.0	3.7	3.2	5.1	5.0	5.7	4.3
	Delta as a %	18%	−49%	−40%	−40%	−23%	1%	−14%	−27%	−20%	−11%	−21%
	*AOK SHI fund 2006*	*3.6*	*4.1*	*4.5*	*5.1*	*5.3*	*2.3*	*3.4*	*4.0*	*4.6*	*5.3*	*3.9*
	*Delta 2006 as a *%	*−22%*	*−39%*	*−31%*	*−35%*	*2%*	*63%*	*−21%*	*−7%*	*−14%*	*−3%*	*−13%*
**Physician visits**	KORA-Age	9.2	9.5	10.7	12.7	13.2	9.8	10.7	11.4	14.4	13.0	10.9
	AOK SHI fund 2009*	29.0	36.3	38.7	39.8	43.0	33.0	36.2	39.2	37.4	40.5	35.9
	Delta as a %	−68%	−74%	−72%	−68%	−69%	−70%	−70%	−71%	−62%	−68%	−70%
	*AOK SHI fund 2006*	*28.2*	*33.3*	*36.0*	*37.1*	*38.4*	*30.1*	*32.3*	*33.8*	*36.2*	*38.2*	*33.1*
	*Delta 2006 as a *%	*−67%*	*−71%*	*−70%*	*−66%*	*−66%*	*−68%*	*−67%*	*−66%*	*−60%*	*−66%*	*−67%*
**Number of drugs for treatment of chronic conditions**	KORA-Age	1.9	2.2	2.8	2.9	2.9	1.9	2.2	2.6	3.0	3.1	2.4
	AOK SHI fund 2009*	2.3	2.5	2.8	2.8	3.3	2.3	2.5	3.0	3.1	3.5	2.7
	Delta as a %	−17%	−9%	0%	4%	−12%	−16%	−9%	−12%	−5%	−10%	−9%
	*AOK SHI fund 2006*	*1.9*	*2.2*	*2.3*	*2.4*	*2.4*	*1.9*	*2.2*	*2.5*	*2.7*	*2.6*	*2.2*
	*Delta 2006 as a *%	*2%*	*2%*	*21%*	*24%*	*18%*	*3%*	*2%*	*6%*	*12%*	*21%*	*8%*

*Extrapolated from 2006 data.

### Outpatient services

As presented in Table 3, female KORA-Age participants reported slightly more physician contacts than their male counterparts. Within the extrapolated AOK data there was no such definite trend. Irrespective of age group and sex the self-reported visits reached only 30–40% of contacts as documented within claims. Standardized for the Bavarian resident population, KORA-Age participants incurred about 10.9 physician visits per capita, but AOK insurants incurred three times as many visits, namely 35.9.

### Drugs for chronic conditions

Both KORA-Age and extrapolated AOK data suggested that older age was associated with more intense pharmacological treatment of chronic conditions. Standardized for the Bavarian resident population, we observed that the number of prescribed drugs for the treatment of chronic conditions in the KORA-Age study was about 9% lower than among the AOK insurants.

### Sensitivity analysis for KORA-Age participants insured by AOK

From the 4,127 KORA-Age participants, 1,105 (26.8%) were insured by AOK at baseline. If utilization rates were calculated for this subgroup only, estimates were slightly higher than those for all SHI insurants (entitlement to LTCI: 6.3%; mean inhospital days: 3.5; mean physician visits: 10.9; mean number of drugs: 2.6).

### Comparison between individuals with and without diabetes

In the KORA-Age study, the age- and sex-adjusted mean number of hospital days in individuals with drug-treated diabetes (4.71; 95% CI: 3.52–5.90) was 1.49 times higher than in individuals without diabetes (3.16; 95% CI: 2.68–3.64). For the mean number of physician visits, this ratio was 1.37 (diabetes: 14.24; 95% CI: 13.00–15.49; no diabetes: 10.35; 95% CI: 9.85–10.85).

Although the mean level of utilization was higher in the AOK sample, the relative ratio of utilization between individuals with and without diabetes was quite similar to the KORA-Age estimates. The mean number of hospital days for individuals with diabetes (5.54; 95% CI: 5.19–5.88) was 1.56 times higher than for individuals without diabetes (3.55; 95 CI: 3.37–3.74). For the number of physician visits, the ratio was 1.40 (diabetes: 45.42; 95% CI: 44.67–46.17; no diabetes: 32.39; 95% CI: 31.99–32.80).

## Discussion

Estimates of health care utilization from population surveys are subject to non-response bias and recall bias. In order to assess the extent of recall bias, it is necessary to link self-reported data from survey participants with their individual claims data, whereas the investigation of non-response bias requires the comparison of claims data from participants and non-participants. In Germany, as in many other countries, individual linkage is costly and challenging. Moreover, obtaining data from non-responders without their consent is prohibited by law. The alternative chosen in the current study was to compare survey-based estimates with external data. Thus, we could only examine whether estimates obtained from the survey over- or underestimate the ‘true’ amount of health care utilization in the population, but could not exactly determine the mechanisms behind these discrepancies.

Our study compared estimates obtained from the KORA-Age study with official statistics on LTCI and with estimates from an independent SHI sample. As the KORA study region may not be fully representative of Germany as a whole, we compared the survey data with regional (i.e. for the state of Bavaria) instead of national official statistics. Also, the claims data refer to the market-leading Bavarian SHI fund and may therefore be more suitable for comparison than data from a nationwide SHI fund. It should be noted that the claims data and the survey data refer to different regions in the state of Bavaria. However, the comparison of demographics and overall health care utilization rates suggested that there were no fundamental differences between the two regions and Bavaria as a whole. Thus, it can indirectly be concluded that there is also no difference between both regions.

Our results showed that the KORA-Age study underestimated the mean utilization of long-term care, outpatient and inpatient services, whereas the estimated utilization of pharmaceuticals was very close to the comparative claims data. Although the design of our study does not allow disentangling recall bias from non-response bias, the discrepancies in some domains may be more likely to result from poor recall than non-response, and vice versa. For example, one can assume a rather high recall of long-term care entitlement because care level assignment requires a standardized medical examination by trained physicians from the Medical Review Board of the SHI funds performed either at the applicants’ home or in the nursing home [35]. Thus, the small proportion of individuals entitled to long-term care services in KORA-Age is probably caused by a higher rate of non-participation among these subjects, which apparently in particular affects higher care levels. The true proportion was underestimated, although we also arranged home visits and proxy interviews in order not to exclude those needing a high level of care, especially those living in nursing homes. In total, 27 individuals among the KORA-Age participants lived in a nursing home, and data from 22 of them came from a proxy interview with either a family member or a professional care giver.

Regarding the estimation of inpatient services, the picture is less clear. Although several studies observed that individuals have a good recall of major and rare events such as hospitalizations [2,7], patients may not accurately recall the length of hospital stay [36].

Consistent with previous findings, the self-reported number of physician visits in our study underestimated the mean utilization rates documented in the claims data [37-39]. Research by Glandon et al. [40] focusing on the older population found that individuals tended to underreport the volume of outpatient services by 10.4%. This underreporting level was much less pronounced than in our study, which may be due in part to the different levels of utilization. Previous work has shown that the extent of underreporting increases with increasing utilization [37]. Whereas the mean number of physician visits in a 6-month period was 3.38 in the study by Glandon et al., the corresponding number in the AOK claims data was five times higher. Owing to the limited interview time, the KORA-Age study only used a single question to assess outpatient services. Individuals may better recall past utilization if they are asked to report separately the visits to general practitioners and to different medical specialists. Unpublished results from a former KORA study in the age group between 65 and 85 years showed that respondents reported on average a 1.5 times higher number of physician visits if questions distinguished between 15 different medical specialities. However, even when multiplying the estimates of physician visits from the KORA-Age sample by 1.5, the underestimation is still substantial compared with claims data. This may partly be explained by the fact that respondents may forget to report physician visits that do not require direct patient–physician interaction such as picking up a repeat prescription.

Comparing survey and insurance data on drug use, we found similar estimates on the overall level as well as within age groups. As our survey question covered current medicine, recall bias can be expected to be absent [29]. Also, the question was part of the postal questionnaire so that participants were asked to list not only the names of currently used drugs but also the PZN written on the package. This allowed a thorough identification and a precise coding according to the ATC code. Therefore, one can assume a high validity of self-reports in our study. This is in line with previous research suggesting high agreement between self-reported use of drugs to treat chronic conditions and individually linked insurance data [29,30]. As a consequence, it is likely that the small differences between survey and insurance observed in our data are mainly due to non-response bias.

Previous research on the impact of non-response on health care utilization estimates showed no consistent results. Although according to some studies non-respondents tend to have higher health care utilization rates than responders, especially in the older population [8,9], other studies observed no substantial difference [16].

There are considerable discrepancies between self-reports and claims data when estimating mean utilization levels of inpatient and outpatient services. Nevertheless, we found that surveys in the elderly may still validly estimate differences in utilization existing between different groups of individuals. For example, the ratio of mean utilization between individuals with and without drug-treated diabetes in the KORA-Age study was nearly the same as in the claims data. This suggests that estimating ratios instead of absolute differences may be more appropriate in such data. Concentrating on ratios might also enhance comparisons between different countries, where absolute utilization rates are expected to differ because of differently designed health care systems.

Our study had some limitations. AOK Bavaria is the leading SHI fund in Middle Franconia; however, its clientele may not be representative of all SHI insurants in the district. In particular, it is known that for historical reasons, AOK insures a comparatively high quota of low-income individuals [41]. As a positive association between economic situation and health status is generally accepted, it is possible that estimates based on the AOK sample overestimate the true average health care consumption in the population. For example, we observed that the proportion of individuals entitled to LTCI services was slightly higher compared with the official statistics. Also, our sensitivity analysis showed that KORA-Age participants insured by AOK had a slightly higher use of health services than participants insured by other SHI funds. However, it must be noted, that due to the industrial history of the KORA study region, many people are enrolled in company health insurance funds. Accordingly, only about one in four KORA-Age participants was insured by AOK. As a consequence, estimates from the sensitivity analysis have more uncertainty, and this holds especially for the entitlement to LTCI, where cell counts for age and sex groups were very small. Also, it must be noted that we used the information on health insurance funds from the baseline surveys, so that some participants might have switched their sickness fund in the meantime. However, there is some evidence from a former KORA study that this does not apply to middle-aged and older age groups to any great extent [42].

Although differences between AOK and other SHI funds with regard to the prevalence of chronic diseases have been reported in the literature [43,44], published age- and sex-specific health care utilization rates from another large German SHI were very close to those of AOK Middle Franconia [25,26].

As a further limitation, claims data referred to 2006 and KORA-Age data mainly to 2009. As published official statistics indicated an increase in utilization in some domains during this 3-year period, we applied specific extrapolation methods in order to achieve comparability. Although the method used to extrapolate drug utilization was based on very detailed official statistics about the specific increase in each age and ATC group, we did not have comparable information for the inpatient and outpatient domains. Thus, we assumed constant annual growth rates and extrapolated the increase between 2006 and 2007 observed in the claims data for each sex and age group. However, this rough calculation cannot take into account external events such as changes in the legal framework that may have occurred in the meantime. However, the comparison with unextrapolated 2006 data also revealed a considerable degree of underreporting in the survey.

## Conclusion

In conclusion, our findings suggest that survey estimates based on self-report tend to underestimate the true level of health care utilization in the older population. This especially affects the frequency of physician visits and the entitlement to long-term care services. In contrast, the assessment of drug consumption by postal self-report questionnaires yields similar estimates to the analysis of comparative insurance claims data. In order to disentangle recall from non-response bias, additional direct validation studies linking self-reported and claims data at an individual level are urgently needed. In the long run, corresponding analyses will allow the calculation of age-and sex-specific correction factors.

## Competing interests

The authors declare that they have no competing interests.

## Authors’ contributions

MHu performed the analysis of the KORA-Age data and wrote the majority of the manuscript. LS conducted the claims data analysis and drafted the corresponding sections of the manuscript. MHe was involved in the coordination of the KORA-Age study and prepared the drug data for analysis. AP and RH are principal investigators in the KORA-Age study. RH devised the concept of the paper. All authors (MHu, LS, MHe, AP, RH) critically revised and approved the final manuscript.

## Authors’ information

The KORA study group consists of A Peters (speaker), R Holle, C Meisinger and their coworkers, who are responsible for the design and conduct of the KORA studies.

## Pre-publication history

The pre-publication history for this paper can be accessed here:

http://www.biomedcentral.com/1472-6963/13/1/prepub
